# Contemplating the Impact of the Moderators Agency Cost and Number of Supervisors on Corporate Sustainability Under the Aegis of a Cognitive CEO

**DOI:** 10.3389/fpsyg.2020.00965

**Published:** 2020-05-27

**Authors:** Muddassar Sarfraz, Ilknur Ozturk, Syed Ghulam Meran Shah, Adnan Maqbool

**Affiliations:** ^1^School of Management, Guangdong University of Technology, Guangzhou, China; ^2^Faculty of Economics and Administrative Sciences, Cag University, Mersin, Turkey; ^3^Department of Management Sciences, Government College University, Lahore, Pakistan; ^4^Department of Management Sciences, Khwaja Fareed University of Engineering and Information Technology, Rahim Yar Khan, Pakistan

**Keywords:** entrepreneurship psychology, cognitive CEO, agency cost, supervisors, corporate sustainability

## Abstract

The social cognitive theory emphasizes the risk-taking behavior of an entrepreneur, which leads him to make the right decisions. In this regard, this study formulates the concept of the cognitive CEO through the DAE statistical technique. Specifically, CEO attributes such as CEO age, CEO compensation, CEO tenure, goodwill, and the number of CEO-attended meetings are used as inputs that influence the intangible assets, the output. Chinese SMEs have been selected for empirical analysis for the years 2014–2018. The empirical results reveal that having a cognitive CEO augmented corporate sustainability, while agency cost and the number of supervisors strongly diminished corporate sustainability. Meanwhile, high earnings per share and high total assets are vehicles for maintaining the sustainable growth of firms. Additionally, it is indicated that firms with a loan burden cannot maintain sustainable corporate growth. Lastly, the execution of 2SLS and GMM instrumental regressions authenticate the veracity of results.

## Introduction

An effective corporate governance mechanism assists in mitigating agency conflicts, which ultimately invigorates sustainability (Hussain et al., 2018). Suggestively, under the aegis of agency theory and stakeholder theory, good corporate governance is responsible for firms’ sustainable growth while securing the rights of minority shareholders (Jo and Harjoto, 2011; Crifo et al., 2019). Corporate governance describes the mechanism that forces the upper echelon under the leading role of CEO (Wirtz, 2011) to execute strategies suitable for coping with the dynamic business environment. Convincingly, distinct aspects of CEO attributes have been suggested to influence the firm’s growth (Kim et al., 2016; Ou et al., 2018; Park et al., 2018) and ultimately to affect corporate sustainability. However, the cognition of the CEO must still include contemplation with deep insight, which can be conducive for the firm’s sustainable growth.

Cognitive psychology has revealed that adopting cognitive-style strategies assists CEOs in accumulating the information and knowledge that orientate the organization toward innovative performance (Carnabuci and Diószegi, 2015; De Visser and Faems, 2015). Meanwhile, the cognitive style of the CEO also supports him/her in decision making, which can determine the future sustainable growth of the firm (Gallén, 1997). The cognitive-style CEO executes strategies that not only resolve problems but invigorate the firm’s sustainable growth (Torrence and Connelly, 2019). Some psychological factors are also associated with CEO motivation and cause him/her to elevate the current position of the organization. In this regard, CEOs endeavor to mitigate the agency cost problem (Boivie et al., 2011). However, it would be interesting to analyze whether the presence of a cognitive CEO mitigates the agency cost problem. Therefore, this study has contemplated this issue empirically.

Doubtless, cognition assists the individual in making the right decision via synchronization of brain functionality (Barsalou, 2014). Cognition has been demonstrated as a vehicle that orientates employees toward innovation and causes a boost to the firm’s growth (Chen et al., 2019). Psychologists have argued that cognition can be assessed through some specific individual characteristics (Zor et al., 2019). They have stated that an individual with the traits of extraversion may have a long memory and that an individual with the characteristic of openness will have excellent cognitive skills (Curtis et al., 2015). In this regard, the concept of the cognitive CEO, the existence of which affects a firm’s sustainable growth, has been formulated.

Prior study has found that corporate CSR sustainability and economic sustainability escalate corporate sustainability (Yang et al., 2019). Chinese firms have distinct characteristics, which is why they have been selected for empirical analysis. Corporate governance is novel among Chinese firms. CEOs are chosen, and government intervention is high (Jiang and Kim, 2015). Further, firms are categorized into SOEs and non-SOEs, which are also controlled via excessive government surveillance through CEOs (Wu et al., 2018; Grøgaard et al., 2019). Despite experiencing excessive control by the government, SOEs are suffering from the agency cost problem (Jia et al., 2019). However, the board size among Chinese firms mitigates the risk (Huang and Wang, 2015), which signifies the role of independent directors and supervisory board members. Though previous research has examined the impact of independent directors, it has neglected the effect of the number of supervisors; this study has filled this gap through contemplating the role of the number of supervisors as a moderator.

The corporate governance mechanism has been ameliorated through the promulgation of specific rules by the CSRC^1^, which have made it compulsory to have a particular number of independent directors. Chinese firms also allocate supervisory board members to enhance the efficiency of the upper echelon. In this regard, this study has analyzed the role of supervisory board members who can assist the cognitive CEO while enhancing corporate sustainability. Significantly, Chinese SMEs are contributing considerably (60%) to GDP (Zheng et al., 2007). Hence, empirical analysis of cognition among CEOs of SMEs is extremely pertinent.

Our study contributes in the following ways. Firstly, sustainability is measured through secondary data variables. Secondly, the cognitive CEO^2^ is formulated through execution of the DAE statistical technique. We select those variables that illustrate cognition. Thirdly, analysis is performed of whether the number of supervisors acts as a moderator that can influence sustainability. Fourthly, agency cost is also considered as a moderator that affects sustainable growth. Meanwhile, this study also makes a theoretical contribution to the agency cost theory perspective, emphasizing that cognition is useless in the presence of the agency cost problem. Lastly, 2SLS instrumental regression has been executed to authenticate the veracity of the results.

## Theoretical Framework and Formulation of Hypotheses

Prior studies have enunciated on the innovative capabilities of SMEs (Newman et al., 2015; Chung and Tan, 2017; Gentile-Lüdecke et al., 2019; Mei et al., 2019; Xiang et al., 2019), but few studies have revealed the effect of corporate governance on SME performance (Hsu et al., 2013; Shapiro et al., 2015; Ge et al., 2019; Bauweraerts, 2020). Despite this enormous body of research, the cognition of the CEO has been little explored, though this can boost the firm’s growth and maintain sustainable corporate growth. Specifically, cognition among the upper echelon enhances their ability to comprehend problems with deep insight (Acedo and Florin, 2006), and cognition also assists in gathering information that allows even an entrepreneur to confront the uncertainties that firms face. Entrepreneurial cognition emphasizes individual traits, which boost the entrepreneurial process (Chen et al., 2015).

Accordingly, cognitive styles elaborate on organizational or individual behavior. Cognition has no interconnection with intellectual ability, so its effectiveness can be a two-edged sword (Armstrong et al., 2012). Cognition among managers can boost the dynamic capabilities that are ultimately conducive to the growth and sustainability of firms (Helfat and Peteraf, 2015). Similarly, (Bromiley and Rau, 2016) have demonstrated that cognitive behavior among the top management team orientates them toward enhancing the firm’s growth.

Among Chinese firms, specific CEO attributes have been signified to be vehicles for the firm’s growth and sustainability (Wei and Ling, 2015; Shahab et al., 2020). The specific attributes of board members also enhance the firm’s growth (Bo et al., 2016). However, research has shed light on the effect of CEO attributes on CEO cognition while boosting the firm’s growth (Liu et al., 2018). A recent study (Li et al., 2020) argues that a cognitive CEO is beneficial for the firm’s performance while having an orientation toward CSR disclosure. To summarize, a cognitive CEO endeavors to accelerate the firm’s growth. Hence, our first hypothesis can be formulated as follows:

**H1:** *A cognitive CEO boosts corporate sustainability in Chinese firms*

The existing literature has signified that specific CEO attributes have a positive relationship with the agency cost problem (Page, 2018). However, some studies have found that CEO succession exacerbates the agency cost problem (Chen et al., 2007). Chinese firms, specifically state-owned enterprises, have been criticized for suffering from the agency cost problem (Huang et al., 2011). It has been shown that agency cost negatively impacts a firm’s growth (Firth et al., 2019). Though good corporate governance can reduce the agency cost (Xiao and Zhao, 2014), Chinese firms are still confronting this problem due to weak corporate governance. It has been found that a CEO with strong discretionary power aggravates the agency cost problem in SMEs (Huang et al., 2016). Specifically, research has revealed that firms adopting CSR activity suffer from the agency cost problem (Lee and Lee, 2019). (Li et al., 2020) have demonstrated that a cognitive CEO augments the firms’ growth and invigorates CSR activity. Therefore, it can be inferred that the agency cost problem as a moderator should deter sustainable corporate growth, even under the auspices of a cognitive CEO. Hence our second hypothesis can be described as follows:

**H2:** *Agency cost as a moderator under the aegis of a cognitive CEO deters corporate sustainability*

SMEs deliberately endeavor to downsize employees and enhance the efficiency of workers so that maximum output may be gained (Newman and Sheikh, 2012). Hence, the role of supervisors is essential, because some research has unveiled that communication with the supervisors elevates the satisfaction level of employees (Gillet et al., 2013), which ultimately escalates sustainable growth. Chinese firms have enhanced their corporate governance mechanism. To improve the efficiency of corporate governance, the CSRC has emphasized the allocation of a specific number of independent directors (Wang et al., 2019). (Ran et al., 2015) pointed out that particular attributes of supervisory board members intensify the quality of accounting. Further, it has been shown in the extant literature that supervisory board members boost the efficiency of corporate governance (Haß et al., 2016). Conversely, it has been demonstrated that supervisory board members represent the specific stakeholders (Kocmanova et al., 2011) while embodying agency cost, which can impede the firm’s growth. Keeping this view, it can be conjectured that supervisory board members should play a vital role in maintaining sustainable growth. However, SMEs are alleged to be riskier organizations (Luo et al., 2018), so there is a chance that having several supervisors will have a negative effect on a firm’s sustainability. Logically, this paves the way toward the third hypothesis:

**H3:** *Supervisory board members as a moderator enhance the firm’s sustainability asymmetrically under the auspices of a cognitive CEO*

## Data Collection and Measures

We have accumulated data on Chinese SMEs listed on both the Shenzhen and Shanghai stock exchanges for the years 2014–2018. The CSMAR and Wind data sources have been preferred for the collection of data following a prior study related to Chinese firms. The independent variable “Cognitive CEO” has been formulated via the DAE statistical technique (Demerjian et al., 2012). Prior research showed that cognition is an ability that can be excavated through previous experiences (Chen et al., 2019) and that it can be illuminated through motivation and knowledge. In this regard, “Cognitive CEO” has been formulated via six variables (among them, five are inputs and one is an output). “CEO tenure,” “CEO compensation,” “Goodwill,” “CEO age,” and “number of CEO-attended meetings” are input variables, whereas “Intangible assets” is an output variable. Mathematically:

(1) $${\mathrm{CGCEO}}_{\mathrm{it}}=\sum_{\tau =1}^{j}l_{\tau }x_{\tau m}/\sum_{\tau =1}^{p}k_{\tau }y_{\tau m},\text{where }\tau =1,\ldots ,n$$

where “p” is inputs and “j” is outputs, which signifies that the cognition ability of the CEO will boost the intangible assets of the firm.

The dependent variable “sustainability” indicates the organizational performance, which has been measured through the proxies sustainable growth and management growth^3^. The variables “LNTA” (total assets), “EPS” (earnings per share), “Firm age,” “LNEMP (firm size), and leverage are interlinked with performance (Huang and Wang, 2015), so these variables have been embedded in the panel regression (Li et al., 2020). Corporate governance is quite novel among Chinese firms. The CSRC^4^ has recently imposed the rule that a specific number of independent directors is required, which is why we have preferred the variable “INDDIR” (number of independent directors). Chinese firms are distinctively categorized into SOE and non-SOE. Therefore, “SOE” has been endorsed as a dummy variable. The variable “Dual” has also been included in the panel regression, which indicates whether a CEO holds two offices^5^ or not while controlling the upper echelon intensively, which can act as a two-edged sword. Specifically, the moderator agency cost has been measured through a proxy (operation ratio) (Ang et al., 2000). The number of supervisors has been endorsed as a moderator. The role of supervisory board members is to enhance the efficiency of corporate governance, and they increase the firm’s growth (Haß et al., 2016). Therefore, supervisors can also assist in maintaining sustainability, which is why their effectiveness as a moderator has been analyzed in the empirical analysis.

## Empirical Models

The panel regression technique has been used to perform the empirical analysis. Through confirmation by the Hausman test, a fixed effect has been selected. The existing literature recommends making interpretations based on the results of 2SLS regression, which is authentic and reliable (Larcker and Rusticus, 2010). Hence, the results of 2SLS instrumental regression have been shown in Tables 3, 4. “Technical Cognitive CEO”^6^ has been endorsed as an instrumental variable. The mathematical formula of the panel regressions is given below.

(2) $$\begin{array}{rl} Sustainability_{it} & =\beta_{0}+\beta_{1it}CGCEO_{it}+\beta_{2it}\;LNEMP_{it}\\ & \;+\beta_{3it}\;SOE_{it}+\beta_{4it}Fage_{it}+\beta_{5it}INDDIR_{it}\\ & \;+\beta_{6it}lnTA_{it}+\beta_{7it}Leverage_{it}+\beta_{8it}EPS_{it}\\ & \;+\delta_{it}Industry+\vartheta_{it}year+\varepsilon_{it} \end{array}$$

(3) $$\begin{array}{rl} Sustainability_{it} & =\beta_{0}+\beta_{1it}\left(CGCEO_{it}*Agency\;cost_{it}\right)\\ & \;+\beta_{2it}\;LNEMP_{it}+\beta_{3it}\;SOE_{it}\;+\;\beta_{4it}Fage_{it}\\ & \;+\beta_{5it}INDDIR_{it}+\beta_{6it}lnTA_{it}+\beta_{7it}Leverage_{it}\\ & \;+\beta_{8it}EPS_{it}+\delta_{it}Industry+\vartheta_{it}year+\varepsilon_{it} \end{array}$$

(4) $$\begin{array}{rl} Sustainability_{it} & =\beta_{0}+\beta_{1it}\left(CGCEO_{it}*NSUPV_{it}\right)\\ & \;+\beta_{2it}\;LNEMP_{it}+\beta_{3it}\;SOE_{it}\;+\beta_{4it}Fage_{it}\\ & \;+\;\beta_{5it}INDDIR_{it}+\beta_{6it}lnTA_{it}+\beta_{7it}Leverage_{it}\\ & \;+\beta_{8it}EPS_{it}+\delta_{it}Industry+\vartheta_{it}year+\varepsilon_{it} \end{array}$$

In eq. (3) the interaction term “*CGCEO*_*it*_∗*Agency cost*_*it*_” demonstrates the impact of agency cost as a moderator, whereas in eq. (4), the interaction term “*CGCEO*_*it*_∗*NSUPV*_*it*_” signifies the role of supervisors as a moderator. Meanwhile, “δ_*it*_*Industry and* ϑ_*it*_*year*” represents the industry dummy variable and year dummy variable, respectively.

## Empirical Results

Table 1 presents the descriptive statistics while revealing the standard deviation, mean, and maximum and minimum values of the variables. The abbreviations LNEMP, SUSGR, MNGR, NSUPV, CGCEO, and INDDIR indicates the number of employees, sustainable growth, management growth, number of supervisors, cognitive CEO, and independent directors, respectively.

**TABLE 1:** Descriptive statistics.

Variables	Obs	Mean	Std. Dev.	Min	Max
INDDIR	2689	3.802157	1.248622	2	13
NSUPV	2689	6.843064	2.632202	3	23
Leverage	2316	0.4952768	0.2101763	0.016418	1.222597
LNTA	2684	19.31819	7.836476	13.87156	28.71384
LNEMP	2686	6.718774	2.968605	1.207341	11.59988
SOE	2618	0.5244069	0.5685188	0	11.85493
Dual	2648	0.3043807	0.4602315	0	1
Fage	2689	9.176091	4.420668	2	29
Agency cost	2673	222.916	5.6777	179.07179	2397
CGCEO	2666	222.167	6.897	176.3677	2453
SUSGR	2689	0.0010578	2.347214	−7.06652	52.07452
MNGR	2646	0.3593005	3.592209	−0.913353	118.6476

Table 1 reveals the number of observations, which are almost the same, except for leverage, which has fewer observations than the other variables. The standard deviation for “CGCEO” is high because of the different values of five input variables. However, these variations are acceptable for empirical analysis.

Table 2 illustrates the correlation among independent variables. The maximum correlation value is “0.4437” between “LNTA” and “LNEMP,” which is also acceptable. Hence, there is no existence of absolute multicollinearity among independent variables.

**TABLE 2:** Correlation matrix.

Variables	1	2	3	4	5	6	7	8	9	10
1. INDDIR	1.000									
2. CCEO	–0.0264	1.000								
3. EPS	–0.0269	0.0560	1.000							
4. Leverage	–0.0043	–0.0028	0.0180	1.000						
5. LNTA	0.0268	0.0249	0.2620	0.3687	1.000					
6. LNEMP	0.0280	–0.0237	0.1714	0.1671	0.4437	1.000				
7. SOE	–0.0071	0.0395	–0.0019	0.0366	0.0894	0.0664	1.000			
8. Fage	0.0551	0.0141	0.0337	0.1034	–0.1047	0.1131	0.0049	1.000		
9. Agency cost	0.0064	–0.375	0.0227	–0.0168	–0.0088	–0.0069	–0.039	–0.04	1.000	
10. NSUPV	–0.0105	–0.039	0.0430	0.1007	0.3554	0.4329	–0.005	–0.05	–0.00	1.000

Table 2 indicates the correlation among independent variables. The maximum correlation values are “0.4437” and “0.4329,” while the remaining correlation values are lower.

Table 3 shows that having a cognitive CEO has augmented sustainability (from the 1st row, the coefficient values of sustainable growth and management growth are “0.351” and “0.269,” respectively. Cognition acts as a catalyst that accelerates the firm’s growth and assists it in maintaining sustainability. Convincingly, our first hypothesis (H1), which states that a cognitive CEO boosts corporate sustainability, has been supported. Further, “EPS” (earnings per share) also supports sustainability. Logically, the variable “EPS” represents how worthwhile investment would be through the provision of dividends. Thus, a high earnings per share ratio is an excellent indicator of a firm’s sustainable growth. Similarly, “LNTA” (total assets) shows positive significance for sustainability. “Leverage” is negatively significant for sustainability, which signifies that higher leverage values are detrimental to firms’ financial health.

**TABLE 3:** 2SLS regression for cognitive CEO and sustainability.

Variables	SUSGR (1)	SUSGR (2)	MNGR (3)	MNGR (4)
CGCEO	0.351**	0.343**	0.216**	0.269**
	(0.210)	(0.206)	(0.103)	(0.117)
EPS	0.194***	0.196***	0.125*	0.145*
	(0.0549)	(0.0533)	(0.0751)	(0.0873)
Leverage	−0.806**	−0.773**	−0.408*	−0.471*
	(0.314)	(0.312)	(0.202)	(0.261)
LNTA	0.376**	0.390**	0.138***	0.155***
	(0.0601)	(0.0600)	(0.0522)	(0.0583)
SOE	0.164	0.146	–0.155	–0.188
	(0.145)	(0.142)	(0.109)	(0.127)
Fage		0.00121		0.00408
		(0.0134)		(0.0110)
Dual		–0.159		0.00376
		(0.172)		(0.141)
INDDIR	–0.0690	–0.0707	0.0159	0.0203
	(0.0529)	(0.0531)	(0.0412)	(0.0507)
LNEMP	–0.0483	–0.0554	−0.0965**	−0.110**
	(0.0584)	(0.0609)	(0.0427)	(0.0473)
Industry_Dummy_	Included	Included	Included	Included
Year_Dummy_	Included	Included	Included	Included
Constant	3.493	3.481	−5.900**	−7.033**
	(3.669)	(3.665)	(2.301)	(3.116)
Observations	1,405	1,380	1,405	1,385
R-squared	0.147	0.132	0.098	0.090

Standard errors in parentheses: ***p < 0.01, **p < 0.05, *p < 0.1.

Table 3 indicates that CGCEO is positively significant for both “sustainable growth” and “management growth” (0.351 and 0.269, respectively). Additionally, “EPS” and “LNTA” are also positively significant for sustainable growth. Reciprocally, “Leverage” is negatively significant for sustainability (3rd row the coefficient values are “−0.806” and “−0.471,” respectively).

Table 4 shows that agency cost (as a moderator) deters sustainability (in the 9th row of Table 4, the coefficient values of “CGCEO* Agency cost” are “−5.73e-05” and “−2.99e-05”). Therefore, our second hypothesis (H2) has been satisfied comprehensively. The values of coefficients are minuscule. The agency cost problem not only deters firms’ growth, but it is also detrimental for firms’ images, which ultimately creates a hurdle to maintaining sustainable growth. In this regard, even a cognitive CEO cannot perform well in the presence of the agency cost problem. The variables “Leverage” and “LNTA” have affected sustainability asymmetrically (as in Table 3).

**TABLE 4:** 2SLS regression for moderator agency cost between cognitive CEO and sustainability.

Variables	SUSGR (1)	SUSGR (2)	MNGR (3)	MNGR (4)
EPS	0.236***	0.235***	0.0851*	0.0741*
	(0.0264)	(0.0264)	(0.0218)	(0.0223)
Leverage	−0.646***	−0.650***	−0.414**	−0.410**
	(0.173)	(0.173)	(0.228)	(0.227)
LNTA	0.0979*	0.0985*	0.133***	0.125***
	(0.0139)	(0.0139)	(0.0470)	(0.0464)
SOE	0.00553	0.00577	–0.106	–0.125
	(0.0653)	(0.0653)	(0.0846)	(0.0835)
Fage	0.000212	–0.000293	0.00405	0.00280
	(0.00762)	(0.00761)	(0.00946)	(0.00934)
Dual	–0.0455	–0.0445	–0.0541	
	(0.0851)	(0.0852)	(0.109)	
INDDIR	–0.0275		–0.0136	
	(0.0263)		(0.0352)	
LNEMP	–0.0173	–0.0177	−0.102**	−0.0897**
	(0.0308)	(0.0308)	(0.0409)	(0.0400)
CGCEO*Agency	−5.73e-05***	−5.71e-05***	−2.99e-05**	−2.56e-05**
cost	(1.94e-05)	(1.92e-05)	(1.39e-05)	(1.17e-05)
Industry_Dummy_	Included	Included	Included	Included
Year_Dummy_	Included	Included	Included	Included
Constant	−1.445**	−1.527**	−2.249*	−2.154*
	(0.693)	(0.689)	(0.721)	(0.708)
Observations	1,380	1,380	1,290	1,290
R-squared	0.128	0.128	0.092	0.092

Standard errors in parentheses: ***p < 0.01, **p < 0.05, *p < 0.1.

In Table 4, “CGCEO∗Agency cost” is negatively significant for sustainable growth (“−5.73e-05***” and “−2.99e-05**,” respectively). Additionally, “EPS” and “LNTA” have influenced the sustainable growth positively (in the 1st row and 3rd row, the coefficient values are “0.236***,” “0.0851*,” “0.0985*,” and “0.133***,” respectively). “Leverage” is negatively significant for sustainability (“−0.650***” and “−0.414**”).

Table 5 indicates that having several supervisors reduces sustainable growth (in the 1st row of Table 5, coefficient values are −0.000317* and −2.82e-05**, respectively), indicating that our third hypothesis (H3) is approved. Conclusively, one reason that may be behind this result is that their extra involvement through their strict surveillance deters even a cognitive CEO from performing independently. In the presence of independent directors, it is unnecessary to increase the number of supervisors, which ultimately causes an extra burden on firms’ financial statements and leads the way toward a decline in firms’ growth.

**TABLE 5:** 2SLS regression for number of supervisors and sustainable growth.

Variables	SUSGR (1)	SUSGR (2)	MNGR (3)	MNGR (4)
CGCEO*NSUPV	−0.000317*	−0.000321*	−2.82e-05**	−2.32e-05*
	(0.000177)	(0.000183)	(1.25e-05)	(1.26e-05)
EPS	0.197***	0.194***	0.0992**	0.0981**
	(0.0493)	(0.0505)	(0.0344)	(0.0342)
Leverage	−0.553*	−0.510*	−0.415*	−0.432*
	(0.291)	(0.295)	(0.107)	(0.116)
LNTA	0.102**	0.110**	0.143***	0.153***
	(0.0214)	(0.0237)	(0.0469)	(0.0471)
SOE	0.0376	0.0401	–0.0980	–0.102
	(0.109)	(0.110)	(0.0841)	(0.0836)
Fage	–0.00330	–0.00678	0.00272	0.000299
	(0.0129)	(0.0130)	(0.00938)	(0.00913)
Dual	–0.111	–0.0807	–0.0762	–0.0694
	(0.149)	(0.146)	(0.108)	(0.107)
LNEMP	0.264	0.263	−0.0807*	−0.0819*
	(0.161)	(0.164)	(0.0421)	(0.0420)
Industry_Dummy_	Included	Included	Included	Included
Year_Dummy_	Included	Included	Included	Included
Constant	−4.058***	−4.445***	−4.037***	−4.058***
	(1.404)	(1.491)	(0.858)	(0.855)
Observations	1,380	1,390	1,274	1,281
R-squared	1.407	1.407	0.086	0.086

Standard errors in parentheses: ***p < 0.01, **p < 0.05, *p < 0.1.

In Table 5, “CGCEO*NSUPV” is negatively significant for firms’ sustainability (in the 1st row of Table 5, the coefficient values are “−0.000317*” and “−2.82e-05**,” respectively). Further, the variables “EPS” and “LNTA” are positively significant for sustainable growth (in the 2nd and 4th rows of Table 5, coefficient values are “0.197***,” “0.0992**,” “0.110**,” and “0.153***,” respectively). “Leverage” is negatively significant for sustainable growth (in the 3rd row, the coefficient values are “−0.553*” and “−0.432*,” respectively).

GMM instrumental regression has been executed, which signifies the veracity of our previous results. In Table 6, all of the results are similar to the previous Tables 3–5. The first two rows identify the positive relation of cognitive CEO with sustainable growth. In contrast, rows 10th and 11th show the negative associations of the moderators (agency cost and several supervisors), respectively.

**TABLE 6:** GMM instrumental regressions of cognitive CEO and moderators for sustainable growth.

Variables	SUSGR	MNGR	SUSGR	MNGR	SUSGR	MNGR
CGCEO	0.507**	0.529**				
	(0.0143)	(0.0160)				
EPS	0.0757**	0.067**	0.0760**	0.0955**	0.0758**	0.216**
	(0.0484)	(0.027)	(0.0550)	(0.0435)	(0.0551)	(0.107)
Leverage	−0.0823*	−0.504**	−0.0726*	−0.652*	−0.0725*	–0.691
	(0.0473)	(0.295)	(0.0477)	(0.362)	(0.0481)	(0.457)
LNTA	0.0193	0.102	0.0177	–0.0217	0.0182	0.216
	(0.0174)	(0.110)	(0.0150)	(0.548)	(0.0149)	(0.151)
LNEMP	–0.00969	–0.0627	–0.00415	0.0857	–0.00443	–0.0124
	(0.0204)	(0.132)	(0.0136)	(0.473)	(0.0127)	(0.126)
Dual	–0.0485	–0.324	–0.0309	–0.0679	–0.0328	–0.499
	(0.0716)	(0.731)	(0.0394)	(0.158)	(0.0490)	(0.753)
SOE	0.0440	0.201	0.0211	0.0900	0.0193	–0.369
	(0.0597)	(0.714)	(0.0355)	(0.472)	(0.0460)	(0.654)
Fage	–0.00209	0.0394	–0.00499	0.0397	–0.00548	–0.108
	(0.00264)	(0.0381)	(0.0147)	(0.0291)	(0.0177)	(0.263)
INDDIR	–0.00365	–0.0833	0.00219	0.00192	0.00173	–0.107
	(0.0207)	(0.213)	(0.0121)	(0.0847)	(0.0144)	(0.168)
CGCEO*Agency cost			−7.21e-05**	−8.01e-05**		
			(1.36e-05)	(2.84e-05)		
CGCEO*NSUPV					−4.02e-06**	−1.95e-07**
					(2.48e-06)	(1.35e-07)
Industry_Dummy_	Included	Included	Included	Included	Included	Included
Year_Dummy_	Included	Included	Included	Included	Included	Included
Constant	0.603	6.929	–0.176	–0.209	–0.175	–1.465
	(2.468)	(28.02)	(0.417)	(7.831)	(0.422)	(2.487)
Observations	1,449	1,422	1,331	1,305	1,331	1,305

Standard errors in parentheses: **p < 0.05, *p < 0.1.

Table 6 signifies the positive significance of cognitive CEO for sustainability (1st row of Table 6). Additionally, the interaction terms “CGCEO*Agency cost” and “CGCEO*NSUPV” have shown negative significance for sustainable growth. Meanwhile, the variables “EPS” and “Leverage” influence sustainable growth asymmetrically.

## Discussion

The empirical results signify that cognitive CEO is positively significant for corporate sustainability. In Table 3, the coefficients of cognitive CEO are “0.351**” and “0.269**,” respectively, which means that cognitive CEO not only enhances sustainable growth but also tends to augment management growth. These results also support our first hypothesis. Logically, cognition intensifies the ability to make the right decisions in the case of a detrimental situation. The extant literature has already provided evidence that cognition among the upper echelon is a blessing in disguise, as it assists them while escalating the firms’ growth (Bromiley and Rau, 2016). Cognition is a vehicle for invigorating the dynamic capabilities that ultimately (Helfat and Peteraf, 2015) boost the firm’s sustainability. In this regard, our first result has contributed by filling the lacunas within the literature while amalgamating cognitive psychology into entrepreneurship psychology, which argues that cognition does not enhance intellectual ability but rather assists with making the right decision.

The empirical results shown in Table 4 reveal that the coefficients of the moderators are “5.73e-05***” and “−2.99e-05**,” respectively. Though the coefficient values are not very high, their negative sign indicates that due to the presence of agency cost, the cognitive CEO cannot maintain sustainability. Thus, our second hypothesis has also been satisfied. Some of the extant literature has argued that agency cost deters corporate sustainability (Firth et al., 2019). Surprisingly, agency conflict negatively affects the quality of corporate governance (Renders and Gaeremynck, 2012), which indicates that the presence of agency cost will decelerate corporate sustainability.

Table 5 indicates that the moderator “number of supervisors” also diminishes corporate sustainability. The coefficients of having several supervisors are “−0.000321*” and “−2.82e-05**,” respectively. This signifies that our third hypothesis has also been satisfied. Though the extant literature has shown that the communication abilities of supervisors can appease employee dissatisfaction (Gillet et al., 2013), SMEs suffer from economic problems and always prefer to enhance efficiency through having a limited number of workers (Newman and Sheikh, 2012). Therefore, having a large number of supervisors will affect corporate sustainability negatively. To encapsulate, the cognitive CEO escalates corporate sustainability, but agency cost and the number of supervisors decrease corporate sustainability.

## Conclusion

Chinese SMEs play a vital role by contributing more than 60% of the country’s GDP annually. Chinese firms have improved their corporate governance and have been compelled to adopt innovative organizational strategies. Despite these great steps, government intervention is excessive. CEOs are less independent than are CEOs working in western and advanced countries. Therefore, they are bound to be answerable in case of poor performance. However, the working efficiency of Chinese SMEs is very good.

With the emergence of entrepreneurship psychology, new concepts regarding cognition have been analyzed by organizational theorists, who have argued that the cognitive ability of the CEO assists him or her in making decisions that are conducive to maintaining the firm’s sustainability. In this regard, the concept of a cognitive CEO has been formulated based on the variables CEO tenure, CEO age, number of CEO-attended meetings, goodwill, CEO compensation, and intangible assets. The specific attributes signify the cognitive abilities of the CEO, which positively influence corporate sustainability.

Chinese firms are criticized as suffering from the agency cost problem, which has been analyzed as a moderator in this study. The results suggest that the agility of a cognitive CEO is weak in the presence of the agency cost problem. Hence, agency cost as a moderator deters corporate sustainability. Moreover, the number of supervisors also acts as a moderator, diminishing corporate sustainability. Meanwhile, it has also been observed that SMEs that have high earnings per share and high total assets can maintain corporate sustainability, whereas SMEs with high leverage cannot.

The study also signifies some implications for academicians, organizational theorists, and practitioners. Firstly, this research suggests that it would be better to eradicate agency cost in Chinese SMEs; otherwise, the specific attributes of the CEO will be useless for regulating corporate sustainability. Secondly, the presence of independent directors is beneficial, but an increase in the number of supervisors is unnecessary among Chinese SMEs. Reasonably, a large number of supervisors will enhance the cost in SMEs while weakening corporate sustainability. Lastly, SMEs should curtail leverage, which creates a hurdle to maintaining corporate sustainability.

## Study Limitations

Though this study has contributed a lot while introducing the new concept of the cognitive CEO, it still has specific limitations that represent a guide toward future research. Firstly, the impact of a cognitive CEO has been analyzed for SME sustainability. Nevertheless, a prospective study could also contemplate the role of innovation as a moderator between cognitive CEO and firms’ sustainable growth. Secondly, the impact of a cognitive CEO on firms’ cash holdings and earnings management could be analyzed. Lastly, the effectiveness of a cognitive CEO could also be analyzed for the United States or European countries.

## Data Availability Statement

The datasets generated for this study are available upon request from the corresponding author.

## Ethics Statement

Ethical review and approval were not required for the study on human participants in accordance with the local legislation and institutional requirements. Informed consent was inferred through the completion of the study.

## Author Contributions

All authors listed have made a substantial, direct and intellectual contribution to the work, and approved it for publication.

## Conflict of Interest

The authors declare that the research was conducted in the absence of any commercial or financial relationships that could be construed as a potential conflict of interest.

## Footnotes

1 Chinese security Regulatory Commission.

2 (Li et al., 2020) have formulated the concept of the cognitive CEO, considering it a dummy variable, while the current study has signifieds the cognitive CEO though different values attained obtained by the DAE technique.

3 The data of “management growth” and “sustainable growth” is are directly accumulated collected through from the CSMAR data source.

4 The Chinese security and regularity commission promulgated issued the new rule for the amelioration improvement of corporate governance in 2004 (Haß et al., 2016).

5 Among Chinese firms, it is a normal phenomenon to hold two offices, which boosts the firm’s growth (Yang and Zhao, 2014).

6 Argumentatively Arguably, cognition is based on experience and a visionary approach. In this regard, CEO technical education has been embedded in the formula (1) via the DAE statistical technique. CEO education has been specified as a dummy variable following (Sarfraz et al., 2019; Shah et al., 2019a, b).
